# The activation of bystander CD8^+^ T cells and their roles in viral infection

**DOI:** 10.1038/s12276-019-0316-1

**Published:** 2019-12-11

**Authors:** Tae-Shin Kim, Eui-Cheol Shin

**Affiliations:** Laboratory of Immunology and Infectious Diseases, Graduate School of Medical Science and Engineering, KAIST, Daejeon, 34141 Republic of Korea

**Keywords:** Viral infection, Viral hepatitis

## Abstract

During viral infections, significant numbers of T cells are activated in a T cell receptor-independent and cytokine-dependent manner, a phenomenon referred to as “bystander activation.” Cytokines, including type I interferons, interleukin-18, and interleukin-15, are the most important factors that induce bystander activation of T cells, each of which plays a somewhat different role. Bystander T cells lack specificity for the pathogen, but can nevertheless impact the course of the immune response to the infection. For example, bystander-activated CD8^+^ T cells can participate in protective immunity by secreting cytokines, such as interferon-γ. They also mediate host injury by exerting cytotoxicity that is facilitated by natural killer cell-activating receptors, such as NKG2D, and cytolytic molecules, such as granzyme B. Interestingly, it has been recently reported that there is a strong association between the cytolytic function of bystander-activated CD8^+^ T cells and host tissue injury in patients with acute hepatitis A virus infection. The current review addresses the induction of bystander CD8^+^ T cells, their effector functions, and their potential roles in immunity to infection, immunopathology, and autoimmunity.

## Viral infection: Inciting an immunological mob

Immune cells that are non-specifically activated during infection can offer protection, but may also inflict collateral damage on infected patients. T cells normally mount an antigen-specific immune response, but certain T cells can become stimulated during viral infection without selective activation by a particular antigen. Tae-Shin Kim and Eui-Cheol Shin at KAIST in Daejon, South Korea, have reviewed current insights into this ‘bystander activation’ phenomenon. They explore how the immune response to viruses such as influenza and hepatitis A produces molecular signals that induce bystander activation of ‘killer’ T cells. In some scenarios, this leads to stronger immune protection, but these cells can also damage host tissues, or contribute to disease progression. Modulating this nonspecific response could prove valuable in managing the severity of viral disease.

## Introduction

**Fig. 1:**
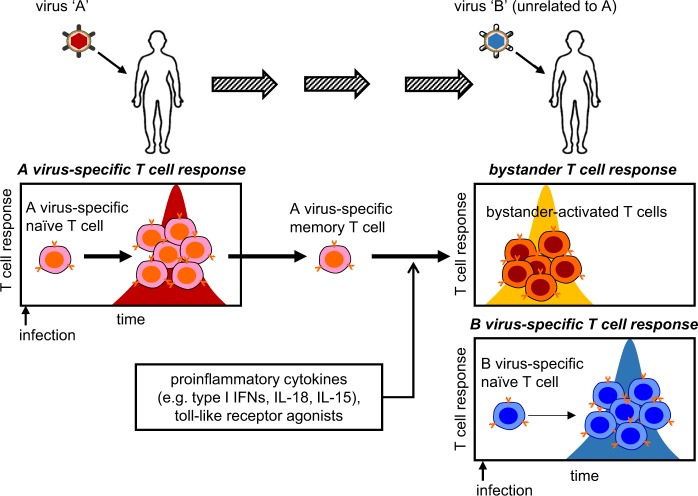
Bystander activation of memory T cells during viral infections. Upon infection with a virus “A,” naive T cells specific for the virus undergo a T cell receptor (TCR)-dependent clonal expansion and then a contraction, leaving memory T cells. Next, infection with an unrelated virus “B” induces TCR-dependent responses as well as TCR-independent “bystander activation” of virus A-specific memory T cells. This bystander response is rapidly induced by cytokines or Toll-like receptor agonists. Type I IFNs, type I interferons; IL-18, interleukin-18; IL-15, interleukin-15

During the course of a viral infection, various immune cells are sequentially activated to eliminate the invading virus. While these immune responses are generally beneficial, they can also cause collateral damage to the host, referred to as “immunopathology”^1^. The nature of the immunopathological response can be significantly impacted by the remnants of the immune response to previous unrelated infections, that is, heterologous immunity^2^. Heterologous immune responses can include both antigen-dependent T cell activation by cross-reactive memory T cells and antigen-independent activation by cytokines (i.e., bystander activation)^3,4^. While intensive studies have revealed the nature and pathophysiological significance of T cell cross-reactivity^2^, relatively little is known about the induction and function of bystander T cells. In this review, we will discuss various aspects of the bystander CD8^+^ T cell response (Fig. 1), including the underlying mechanisms of T cell activation, the pathophysiological impact of activated bystander T cells during infection, and the longer-term clinical implications.

## Bystander activation of CD8^+^ T cells during viral infections

### Acute hepatitis A virus infection

Infection of adults with hepatitis A virus (HAV) can result in acute hepatitis A (AHA) and severe liver injury. It was previously hypothesized that liver injury resulted from an excessive virus-specific T cell response during AHA^5,6^. Consistent with this hypothesis, HAV-specific CD8^+^ T cells were detected by both HLA-A2 tetramer binding and intracellular cytokine staining in acutely infected patients^7^. However, a study using chimpanzees challenged with HAV showed that functional HAV-specific CD8^+^ T cells increased only after viremia and liver injury began to decline^6^.

**Fig. 2:**
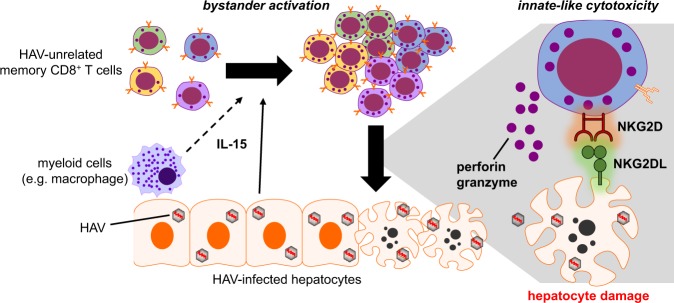
Pathological role of bystander-activated CD8^+^ T cells in acute hepatitis A virus (HAV) infection. During acute hepatitis A, memory CD8^+^ T cells specific for HAV-unrelated viruses undergo IL-15-dependent bystander activation. IL-15 is produced by hepatocytes and possibly myeloid cells in the infected liver. These activated, HAV-unrelated CD8^+^ T cells exhibit “innate-like cytotoxicity” to hepatocytes, which is triggered by the ligation of natural killer cell-activating receptors (e.g., NKG2D) with their ligands

Recently, our group demonstrated that CD8^+^ T cells specific for pathogens other than HAV are activated by a T cell receptor (TCR)-independent but interleukin-15 (IL-15)-dependent mechanism during acute HAV infection^8^ (Fig. 2). These bystander-activated CD8^+^ T cells expressed high levels of cytotoxic molecules (perforin and granzyme B) and natural killer (NK) cell-activating receptors (NKG2D and NKp30) and exhibited innate-like cytotoxicity against hepatocytes. Furthermore, the number of bystander CD38^+^HLA-DR^+^ (activated) CD8^+^ T cells but not that of HAV-specific CD8^+^ T cells was strongly correlated with the level of liver injury during AHA^8^. These activated, HAV-unrelated CD8^+^ T cells were specific for a number of unrelated viruses, including human cytomegalovirus (HCMV), Epstein–Barr virus (EBV), influenza A virus (IAV), respiratory syncytial virus, and vaccinia virus^8^. It is unlikely that these cells were activated by TCR-dependent cross-reactivity given the very small HAV RNA genome (7.5 kb)^5^ and the limited amino acid sequence homology between HAV proteins and epitope peptides used in the tetramer detection of HAV-unrelated viruses^8^. In addition, the significant increase in NKG2D expression on HAV-unrelated memory CD8^+^ T cells compared to that on HAV-specific CD8^+^ T cells in AHA patients further supports bystander activation rather than TCR-dependent activation. Treatment of peripheral blood mononuclear cells from healthy donors with IL-15 increases the level of NKG2D expression in memory CD8^+^ T cells, whereas TCR stimulation with an anti-CD3 antibody or cognate peptide does not. Interestingly, the expression of NKG2D is not significantly increased when the cells are stimulated with both IL-15 and anti-CD3 antibodies^8^, suggesting that NKG2D upregulation on memory CD8^+^ T cells reflects activation by IL-15 in the absence of TCR stimulation^8^. Taken together, these findings provide considerable evidence that the activation of HAV-unrelated CD8^+^ T cells is mediated by an antigen-independent bystander mechanism.

### Hepatitis B virus and hepatitis C virus infections

Nonspecific T cell responses have not been extensively analyzed during hepatitis B virus (HBV) and hepatitis C virus (HCV) infections. Unlike HAV, both HBV and HCV can establish chronic persistent infection in the host^9^. Because virus-specific CD8^+^ T cells become functionally exhausted during the chronic stages of viral infection, it has been postulated that bystander CD8^+^ T cells might contribute to immunopathologic liver injury during chronic HBV or HCV infections^9^.

Maini et al.^10^ examined the frequency and function of HBV-specific CD8^+^ T cells in patients with chronic HBV infection. They unexpectedly found that liver injury was not associated with an increase in the frequency of HBV-specific CD8^+^ T cells. Instead, their data suggested that HBV-unrelated CD8^+^ T cells infiltrating the liver contribute to pathological injury^10^. In support of this idea, Sandalova et al.^11^ have reported that CD8^+^ T cells specific for HBV-unrelated pathogens (HCMV and EBV) were activated in the absence of evidence for HCMV or EBV reactivation in 20 patients with acute HBV infection.

In the case of HCV infection, there are no published reports of bystander activation. However, it has been shown that naive CD8^+^ T cells from patients with chronic hepatitis C exhibited hyperactivation (accompanied by a decreased expression of CD5) and differentiated into memory-phenotype cells^12^. The relationship between this phenomenon and bystander activation remains unknown.

### Human immunodeficiency virus infection

Systemic immune activation, specifically for T and B cells, is one of the hallmarks of untreated chronic human immunodeficiency virus (HIV) infection^13,14^. Although it has been hypothesized that both antigen-dependent and antigen-independent mechanisms mediate HIV-associated immune activation, the exact mechanism remains unclear^13^.

Recent studies have provided evidence for bystander activation during HIV infection. The Oxenius group reported that HIV-1 rebound due to the interruption of antiretroviral therapy (ART) led to the activation and expansion of CD8^+^ T cells irrespective of their antigen specificities^15^. Their data further suggested that myeloid dendritic cell (DC) activation and the resulting IL-15 production drive bystander activation of CD8^+^ T cells during HIV-1 infection^15^. Younes et al.^16^ examined the T cell repertoire of untreated HIV-infected patients and found that the TCR diversity of cycling effector/memory CD8^+^ T cells reflected that of the entire effector/memory CD8^+^ population. The authors concluded that the activation and expansion of the CD8^+^ T cells was driven by nonspecific, bystander activation^16^. In fact, bystander activation of CD8^+^ T cells appears to occur early in HIV infection. During primary HIV infection, activation markers (e.g., CD38 and HLA-DR) are upregulated in the total CD8^+^ T cell population and, more importantly, in the CD8^+^ T cells specific for HIV-unrelated viruses, such as EBV, HCMV, and IAV^17,18^.

Immune activation is an important factor contributing to disease progression during HIV infection. Importantly, bystander activation of CD8^+^ T cells may be one of the drivers of the disease, as a strong correlation between the activation of CD8^+^ T cells and the rate of CD4^+^ T cell loss has been reported in untreated patients^19^. Even in patients undergoing ART, the persistent activation of CD8^+^ T cells was associated with decreased recovery of CD4^+^ T cells^20^ and an increased risk of non-acquired immunodeficiency syndrome (AIDS)-related clinical events^21^. Furthermore, a study conducted in sooty mangabey monkeys offered interesting insight into the role of bystander immune activation in HIV pathogenesis. Sooty mangabeys are natural hosts for simian immunodeficiency virus (SIV) infection, but do not develop AIDS despite a high level of viral replication^22^. This nonpathogenic infection in sooty mangabeys is accompanied by low levels of immune activation compared to that in HIV-infected humans^22^.

### Influenza A virus infection

It is well established that antigen-specific T cells play a central role in controlling IAV infection^23–27^. However, much less is known about the impact of activated bystander memory CD8^+^ T cells and their possible contribution to pathogenesis^28–30^.

Early studies showed that IAV infections draw bystander memory CD8^+^ T cells into the lung airways from the circulation^28,31^. Despite not being specific for IAV, these memory CD8^+^ T cells express strong cytolytic capacity^29,30^. However, it remains unclear whether these recruited bystander cells have an essential role during a primary infection, although they may accelerate the induction of inflammation during a recall response^29,30^. In humans, Sandalova et al.^11^ have shown that memory CD8^+^ T cells specific for HCMV or EBV exhibited an activated phenotype (CD38^+^HLA-DR^+^) during acute IAV infection, although the number of patients analyzed was small. Recent studies have shown that mucosal-associated invariant T (MAIT) cells can also be activated in a bystander manner during influenza infection and participate in protective immune response both in humans and mice^32,33^.

Overall, a significant degree of bystander activation of memory T cells occurs during influenza infection, although its impact on the course of the infection, if any, is not clear. It is also unclear whether bystander-activated CD8^+^ T cells contribute to immunopathology in influenza.

## Factors inducing bystander activation

The cytokines that induce bystander activation generally overlap with those that regulate the activation of antigen-specific CD8^+^ T cells. Specifically, innate inflammatory cytokines seem to be crucial for inducing bystander activation during infection. Pathogen-associated molecular pattern (PAMP) signaling through Toll-like receptors (TLRs) also supplies important signals for bystander activation.

### Type I interferons

Sprent’s group was the first to report the importance of type I interferons (IFNs) in nonspecific T cell proliferation upon viral infection or lipopolysaccharide injection^4,34^. Indeed, type I IFNs may act directly on CD8^+^ T cells and drive the expansion of T cells during infection with lymphocytic choriomeningitis virus^35^. Moreover, an intriguing study on the pathogenesis of HIV infection suggested that type I IFNs are the key drivers of T cell activation and disease progression in patients with persistent HIV infection^36^. Blocking-type I IFN signaling in humanized mouse models of chronic HIV infection results in a reduction in hyperactivation of T cells and their functional recovery^37,38^.

The mechanism of immune bystander activation of memory CD8^+^ T cells by type I IFNs is not known. When antigen-specific memory CD8^+^ T cells are treated in vitro with type I IFNs, they do not exhibit significant functional activation unless also treated with other cytokines, such as IL-18^39^. This result suggests that type I IFNs require additional secondary signals or accessory cells to fully activate bystander T cells. Accordingly, type I IFN signaling may induce IL-15 production by accessory cells^40,41^ and increase T cell responsiveness to IL-18^42^. Both IL-15 and IL-18 are notable mediators of bystander activation.

It is also possible that type I IFNs may have negative effects on the bystander activation of memory T cells during viral infection. A series of experiments performed by Welsh and co-workers^43,44^ revealed that virus-induced type I IFNs mediate rapid attrition of bystander CD8^+^ T cells, especially those with a memory phenotype. This finding suggests that weak rather than strong-type I IFN responses may be optimal for inducing bystander CD8^+^ T cells. In this regard, it is interesting to note that acute HAV infection elicits a relatively weak type I IFN-stimulated gene (ISG) response^45^ and is accompanied by vigorous immunopathology mediated by bystander-activated CD8^+^ T cells^8^.

### Interleukin-18

IL-18, a member of the IL-1 family of cytokines, is one of the most well-characterized cytokines that induce antigen-independent IFN-γ production by effector and memory CD8^+^ T cells during microbial infections^46^. Effector and memory CD8^+^ T cells that are treated with cytokine combinations, including both type I IFNs and IL-18, exhibit an activated phenotype (i.e., CD69^+^) and high levels of IFN-γ production^39^. In addition, studies in vitro and in murine infection models have demonstrated a dramatic synergism between IL-18 and other proinflammatory cytokines (e.g., IL-12, IL-2, IL-15, and IL-21) for inducing antigen-nonspecific IFN-γ production^39,47–49^. Thus, it appears that IL-18 cooperates with a wide range of cytokines in the inflammatory milieu to induce bystander activation of T cells.

IL-18 responsiveness by effector and memory CD8^+^ T cells but not naive CD8^+^ T cells results from selective expression of the IL-18 receptor^48,49^. Recently, Martin et al.^50^ showed that memory CD8^+^ T cells exhibited a gradual reduction in the expression of IL-12 and IL-18 receptors following initial antigen stimulation. Consistent with this finding is a decrease in the ability of the cells to be activated by bystander signals. The opposite is true in the situation when cells are repeatedly stimulated with the antigen.

### Interleukin-15

IL-15, a member of the common γ-chain family of cytokines, is another key factor involved in mediating bystander activation of CD8^+^ T cells in both mice and humans^8,15,16,51,52^. IL-15 has been shown to function in various aspects of lymphoid biology, including the development of NK and invariant NK T (iNKT) cells, the activation of NK cells, and the homeostatic maintenance of memory CD8^+^ T cells (reviewed elsewhere^53^). Importantly, IL-15 can potently induce the activation of murine effector and memory CD8^+^ T cells when synergizing with IL-12, IL-18, or type I IFNs^39^. We and others have shown that memory CD8^+^ T cells from healthy human individuals strongly respond to IL-15 by expressing markers of activation (CD38 and HLA-DR), proliferation (Ki-67), and cytotoxic activity (such as granzyme B)^8,11,16^. In the pathologic conditions such as AHA and untreated HIV-1 infection, the level of IL-15 is elevated in the serum and lymph nodes, respectively. This finding suggests that IL-15 drives bystander activation of CD8^+^ T cells in pathologic situations^8,16^.

**Fig. 3:**
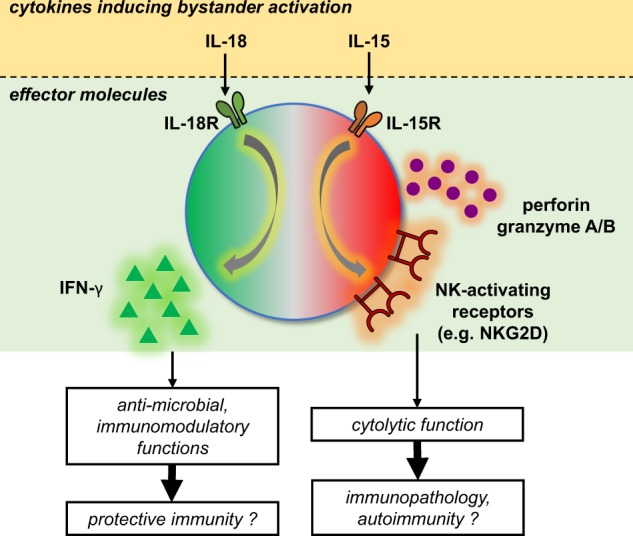
Effector functions of bystander-activated CD8^+^ T cells. Innate inflammatory cytokines, such as IL-18 and IL-15, are crucial for inducing bystander activation during infection. While signaling through IL-18R may contribute to protective immunity via IFN-γ secretion, signaling through IL-15R may lead to immunopathology via NKG2D-dependent cytotoxicity. IL-18R, interleukin-18 receptor; IL-15R, interleukin-15 receptor

The remarkable feature of IL-15 compared to IL-18 is that it confers cytolytic ability on memory CD8^+^ T cells, which can be triggered by NK-activating receptors, such as NKG2D. This phenomenon is referred to as “innate-like (or NK-like) cytotoxicity.” In addition, IL-15 promotes the expression of cytolytic molecules (e.g., granzyme B and perforin)^8,52,54^. A dual control mechanism for the two major effector functions of bystander-activated CD8^+^ T cells, cytokine secretion and cytolytic function, has been suggested by Soudja et al.^52^ whereby IL-18 induces IFN-γ secretion and IL-15 induces the expression of cytolytic molecules (Fig. 3).

Sentinel myeloid cells such as DCs and inflammatory monocytes have been considered the source of IL-15 and IL-18 during viral, bacterial, and fungal infections^52,55^. However, many other cell types, including both hematopoietic and nonhematopoietic cells, can also express IL-15^56^. Indeed, epithelial cells such as enterocytes and hepatocytes have been suggested as the main source of IL-15 in the case of celiac disease and AHA, respectively^8,57^. Further research is needed to determine the relative contributions and functional differences between myeloid and epithelial cells in the production of bystander activation-inducing cytokines during viral infections.

### Toll-like receptors

TLRs are the key receptors in innate immune cells that detect PAMPs and initiate the innate immune response. However, TLRs also function in cells of the adaptive immune system, including T cells^58^. Earlier studies revealed that murine and human effector/memory CD8^+^ T cells express TLR1/2/6 and TLR3, respectively, which function as costimulatory receptors, lowering the threshold for TCR activation^59,60^. More recently, Salerno et al.^61^ discovered that murine memory CD8^+^ T cells can be directly stimulated with the ligands for TLR2 and TLR7 to produce IFN-γ. However, TLR2 or TLR7 stimuli in combination with TCR triggering drive cells to produce IFN-γ, tumor necrosis factor-α (TNF-α), and IL-2^62^.

Given that TLR2, TLR3, and TLR7 react with various types of ligands derived from a wide range of pathogens^63^, these data imply that TLR-mediated bystander activation can occur during various kinds of infections^64^. Indeed, TLR2-mediated bystander activation of T cells has been demonstrated to contribute to the development of arthritis in mice infected with *Borrelia burgdorferi*, a causative bacterial pathogen for Lyme arthritis^65^. However, evidence of TLR-dependent bystander activation is still lacking for viral infections. TLR3 and TLR7/8 recognize double- and single-stranded RNAs, respectively, which are the PAMPs usually associated with viruses^63^. Thus, future studies need to focus on the role of virus-related TLRs in the bystander activation of T cells.

## Effector functions of bystander-activated CD8^+^ T cells

Bystander-activated CD8^+^ T cells share many effector functions with antigen-specific CD8^+^ T cells, such as cytotoxicity and cytokine secretion.

### Cytotoxicity and NKG2D

NKG2D encoded by KLRK1 was first identified in human NK cells as one of the NK cell-activating receptors and was subsequently shown to be expressed by many other lymphoid cells, such as iNKT cells, γδ T cells, and CD8^+^ αβ T cells^66^. In CD8^+^ T cells, it has been suggested that NKG2D mediates a costimulatory function in the presence of TCR engagement^67–70^. Engagement of the TCR and NKG2D results in enhanced cytokine production and proliferation in CD8^+^ T cells with effector^67^ and memory phenotypes^68,69^. The expression of NKG2D ligands is upregulated in multiple tissues during stress conditions, such as viral infection and cellular transformation^71^, and it has been hypothesized that signaling downstream of NKG2D–NKG2DL interactions regulates the activation of antigen-stimulated effector/memory T cells in the local tissue environment^67^.

NKG2D signaling can also elicit cytolytic function in the absence of TCR engagement^72^. For example, freshly isolated CD8^+^ TCRαβ^+^ intraepithelial lymphocytes (IELs) from patients with active celiac disease or IELs prestimulated with IL-15 exhibit NKG2D-mediated cytotoxicity without TCR engagement^67,73^. Moreover, bystander-activated CD8^+^ T cells in patients with AHA exert innate-like cytotoxicity against hepatocytes via a TCR-independent, NKG2D-dependent manner^8^. This effect is reminiscent of what occurs in patients with celiac disease^73^. It is noteworthy that excessive killing of target cells by bystander-activated CD8^+^ T cells may initiate and propagate the cycle of inflammation and immunopathology^74^. The role of NKG2D in mediating the effector function of bystander-activated CD8^+^ T cells has also been demonstrated in mouse models of bacterial and parasitic infections^75,76^.

It should be noted that signaling through the IL-15 receptor induces the upregulation of NKG2D expression^8,54,67,73^. Intriguingly, concurrent TCR activation abrogates IL-15-induced upregulation of NKG2D on the surface of memory CD8^+^ T cells^8^. This result supports the idea that NKG2D preferentially acts in the absence of TCR stimulation. Another important signal that downregulates NKG2D expression during viral infection is type I IFN^77^. Thus, NKG2D expression in bystander-activated CD8^+^ T cells is probably modulated through the balanced action of various proinflammatory cytokines (e.g., IL-15 and type I IFNs) and TCR signaling.

### Interferon-γ

Cytotoxic activity and cytokine production are the major effector mechanisms mediated by CD8^+^ T cells during viral infection^78–80^. The best-known examples of CD8^+^ T cell-derived cytokines are IFN-γ and TNF-α^61,81^. While the expression of both IFN-γ and TNF-α is induced in CD8^+^ T cells stimulated with peptide antigen, only IFN-γ is induced upon treatment with cytokines such as IL-12 and IL-18^81^. Similarly, antigen-experienced murine CD8^+^ T cells stimulated with TLR ligands produce IFN-γ but not TNF-α^61^.

In line with these data, several studies using various infection models (e.g., bacteria, viruses, and parasites) showed that memory CD8^+^ cells underwent bystander activation with rapid upregulation of IFN-γ^47,48,52^. As expected, the induction of IFN-γ in bystander-activated CD8^+^ T cells conferred enhanced control over the challenging bacterial pathogen^48,52^. Although yet to be confirmed, bystander-derived IFN-γ may also have a protective effect against viral pathogens^82^. The protective action of IFN-γ produced by bystander-activated T cells clearly contrasts with the pathological consequences of NKG2D-mediated cytotoxicity observed during AHA in humans or during *Leishmania* infection in a mouse model^8,76^.

A recent paper using high-throughput single-cell analysis of CD8^+^ T cells offered insight into how the same CD8^+^ T cells can exhibit different functional consequences according to the context. When antigen-specific CD8^+^ T cells were stimulated with cognate antigens, they exhibited either cytokine secretion or cytolytic activity (but rarely both), indicating that these two functions are independently regulated^83^. This functional differentiation may also be true of bystander-activated T cells. Indeed, while both IFN-γ secretion and NKG2D-mediated cytolysis are observed in bystander-activated CD8^+^ T cells during *Listeria* infection^48,52^, only NKG2D-mediated cytolysis and consequent immunopathology are noticeable during *Leishmania* infection^76,84^. The factors contributing to this functional difference are currently unclear, but may include the pathogen load, chronicity of inflammation^76^, location of CD8^+^ T cells^84^, and surrounding cytokine milieu^52^.

## Clinical implications of bystander activation

Tough et al.^4^ who first identified bystander activation during viral infection, predicted that the physiological role of bystander activation is to maintain memory CD8^+^ T cells in vivo in the absence of further cognate antigenic stimulation. The hypothesis seemed plausible; however, it has not been demonstrated experimentally. Although bystander-activated CD8^+^ T cells express functional effectors, the precise role in host immunity at the time of infection or thereafter has not been clearly defined.

### Protective vs. pathological role

Bystander activation of T cells during the early stages of infections may contribute to an overall protective immune response. Compared to the antigen-specific T cell response, which takes several days to develop, bystander activation of memory T cells can occur rapidly in response to innate cytokines (e.g., type I IFNs, IL-18, and IL-15), establishing a primary line of defense^4,47–49,52^. Despite lacking specificity for the invading pathogen, these cells may engage an inflammatory process that accelerates immune recruitment to the site and helps to control pathogen loads through the rapid production of IFN-γ, which has direct antimicrobial and immunomodulatory functions^82,85^ (Fig. 3). Indeed, the protective function of adoptively transferred bystander memory T cells was especially evident in IFN-γ-deficient recipient mice^86^.

Perhaps, a more clinically important question is the role of bystander activation in contributing to immunopathology. As described above, bystander-activated T cell-mediated immunopathology is observed in mainly local tissues (e.g., hepatocytes in AHA and skin lesions in *Leishmania* infection) and after sustained inflammation^8,76^ (Fig. 2). These results suggest that bystander-activated CD8^+^ T cells have different phenotypic and functional characteristics depending on their location and duration of exposure to inflammation. More studies are needed to clarify the conditions that induce bystander-activated CD8^+^ T cells involved in immunopathology.

### Implications for autoimmunity and antitumor immunity

What would happen if CD8^+^ T cells specific for self-antigens were activated via a bystander manner during infections? In fact, both microbial infections and bystander T cell activation have long been suggested as contributing factors for autoimmune diseases^14,87,88^. In this regard, a scenario in which bystander activation of T cells triggered by viral infections accelerates the onset of type 1 diabetes has been supported in animal models, although clinical data are lacking^89^. Interestingly, autoreactive T cells are dependent on IL-15 for their maintenance and antigen-independent activation^90^. Furthermore, autoreactive CD8^+^ T cells primed with IL-15 and IL-21 are able to induce disease in a murine model of autoimmune diabetes^91^. Recently, memory CD4^+^ T cells have been shown to undergo bystander activation^92^ and increase the susceptibility of mice to experimental autoimmune encephalomyelitis, a model for multiple sclerosis^93^. In the future, it will be interesting to investigate the relationship between viral infections with strong bystander activation, such as AHA, and the development of subsequent autoimmune complications.

Bystander activation of CD8^+^ T cells may play a role in antitumor immune responses. In mice treated with highly active immunotherapeutic agents, such as a CD40 agonist and IL-2, memory CD8^+^ T cells underwent bystander activation with upregulation of NKG2D and granzyme B^94^. In addition, recent elegant studies have revealed an abundance of intratumoral bystander CD8^+^ T cells without tumor antigen specificity in various types of human cancer, although their roles are not yet clear^95–97^. These findings suggest that bystander CD8^+^ T cells may participate in antitumor immune responses.

## Conclusion and future perspectives

Bystander activation of memory CD8^+^ T cells by cytokine stimulation is an important aspect of immune responses to pathogens. Antigen-independent activation of these T cells may either contribute to protection or initiate aberrant immune responses, such as immunopathology or autoimmunity. Despite ample evidence demonstrating the occurrence of bystander activation during viral infections, its pathophysiological role remains poorly understood.

Many aspects of bystander activation regarding its induction and function have been revealed using animal models. Recently, reported “dirty” mouse models—laboratory mice that have been co-housed with pet store mice or that have undergone sequential infection—are an example of how a host’s infection history can change the immune response in subsequent events and provide a realistic model to examine bystander activation^98,99^.

In the future, we need to consider bystander-activated T cells as a therapeutic target to alleviate severe immunopathology during viral diseases and prevent autoimmunity following viral infections.
